# Improved-high-quality draft genome sequence of *Rhodococcus sp*. JG-3, a eurypsychrophilic *Actinobacteria* from Antarctic Dry Valley permafrost

**DOI:** 10.1186/s40793-015-0043-8

**Published:** 2015-09-03

**Authors:** Jacqueline Goordial, Isabelle Raymond-Bouchard, Jennifer Ronholm, Nicole Shapiro, Tanja Woyke, Lyle Whyte, Corien Bakermans

**Affiliations:** McGill University, 21,111 Lakeshore Rd., Ste. Anne de Bellevue, QC Canada H9X 3V9; DOE Joint Genome Institute, Walnut Creek, CA USA; Altoona College, Pennsylvania State University, Altoona, PA USA

**Keywords:** *Rhodococcus* sp. JG-3, Permafrost, Eurypsychrophile, Dry valleys, Antarctica

## Abstract

The actinobacterium *Rhodococcus sp*. JG-3 is an aerobic, eurypsychrophilic, soil bacterium isolated from permafrost in the hyper arid Upper Dry Valleys of Antarctica. It is yellow pigmented, gram positive, moderately halotolerant and capable of growth from 30 °C down to at least −5 °C. The 5.28 Mb high-quality-draft genome is arranged into 6 scaffolds, containing 9 contigs and 4998 protein coding genes, with 64 % GC content. Increasing the availability of genome sequences from cold-adapted species is crucial to gaining a better understanding of the molecular traits of cold adaptation in microbes.

**Electronic supplementary material:**

The online version of this article (doi:10.1186/s40793-015-0043-8) contains supplementary material, which is available to authorized users.

## Introduction

*Actinobacteria* is a ubiquitous phylum in the biosphere, including many environments that exist predominantly and perennially at sub-zero temperatures (cryoenvironments) such as massive ground ice, polar and alpine saline springs and lakes, cryopegs, and permafrost, where it is often a dominant phylum [1]. The molecular traits which allow *Actinobacteria* to predominate in cryoenvironments remains largely unknown. *Actinobacteria* may be protected in the permafrost environment by cyst-like resting forms or arthrospores, as observed in *Arthrobacter* and *Micrococcus* species isolated from permafrost [2]. It is also possible that dominance of *Actinobacteria* are due to increased viability and activity in this phylum, as *Actinobacteria* that can metabolize at sub-zero temperatures have been found [3, 4]. Though Antarctic permafrost has generally been found to harbor orders of magnitude lower culturable microorganisms (0-10^5^ cells/g) than Arctic permafrost, *Rhodococcus* spp. have been readily isolated from both Antarctic and Arctic permafrost [5]. The genome sequence of *Rhodococcus* sp. JG-3 is also of interest since species within the genus *Rhodococcus* are known to have versatile degradative metabolisms for recalcitrant xenobiotics [6], including the capability to degrade halogenated organics [7], short and long chain alkanes [8], and petroleum hydrocarbons [9]. Several reports have investigated the catabolic potential of *Rhodococcus* spp. for contaminant removal at cold temperatures [8, 10, 11]. The public availability of other mesophillic *Rhodococcus* genomes, in addition to other cryophilic bacterial isolates will enable identification of genes and molecular traits which enable cryophilic organisms like *Rhodococcus* sp. JG-3 to thrive in cold and extreme environments.

## Organism information

### Classification and features

**Fig. 1:**
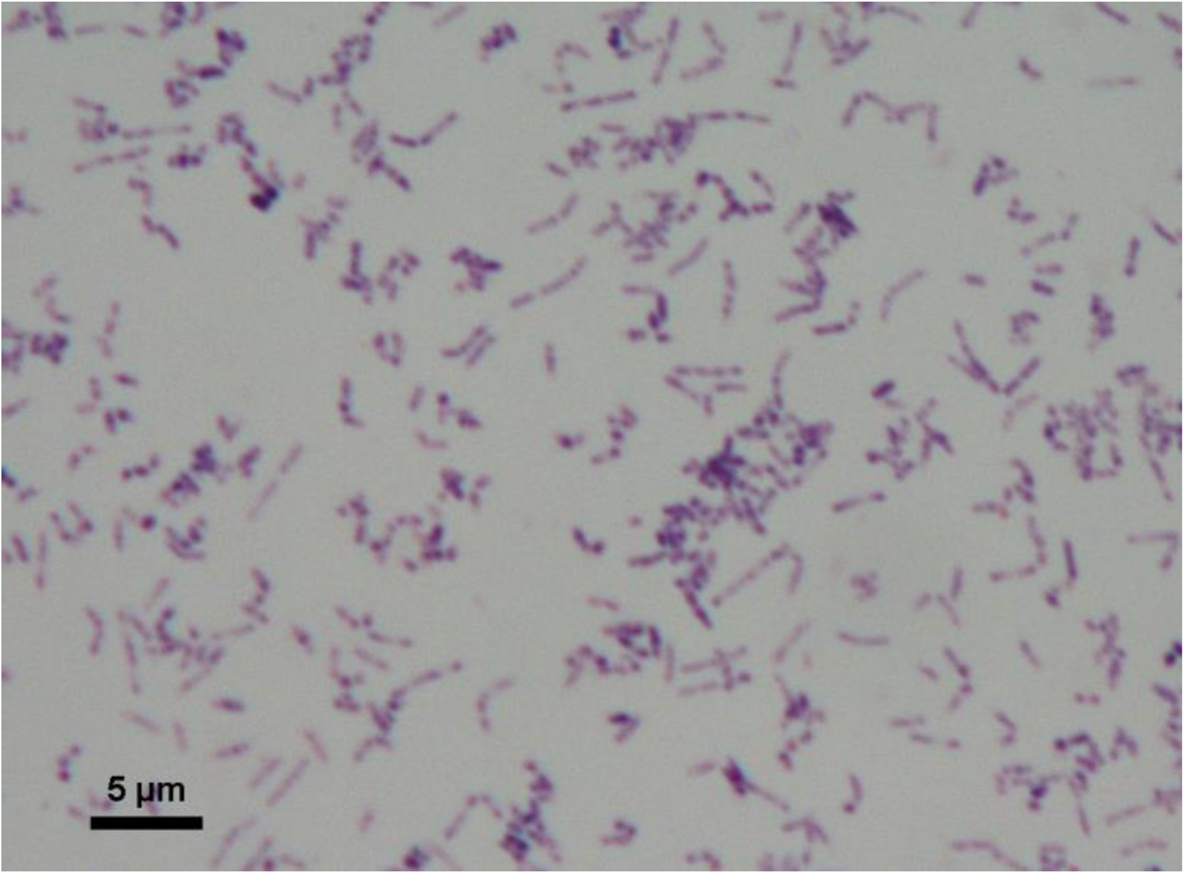
Gram stain of *Rhodococcus* JG-3

*Rhodococcus* sp. JG-3 is a yellow pigmented strain capable of growth from 30 °C down to at least −5 °C. It does not require salt, but is moderately halotolerant up to 7 % NaCl. It is a Gram positive short rod (Fig. 1), and grows well on TSB and R2A media. *Rhodococcus* sp. JG-3 was isolated from University Valley, a small hanging valley (1650–1800 m.a.s.l) above Beacon Valley in the upper elevation McMurdo Dry Valleys, Antarctica. This bacterium was isolated from ice-cemented permafrost soils aged ca. 150,000 years old [12] which experience permanent darkness, hyper oligotrophy (0.013 % total carbon), low water activity (<1 % gravimetric soil moisture content) and constant cold temperature (mean annual soil temperature −24 °C). The classification and general features of *Rhodococcus* sp. JG-3 are summarized in Table 1.

**Table 1:** Classification and general features of *Rhodococcus sp. JG-3* [13]

MIGS ID	Property	Term	Evidence code^a^
	Classification	Domain *Bacteria*	TAS [14]
		Phylum *Actinobacteria*	TAS [15]
		Class *Actinobacteria*	TAS [15]
		Order *Actinomycetales*	TAS [14]
		Family *Nocardiaceae*	TAS [16]
		Genus *Rhodococcus*	TAS [14]
		Species Rhodococcus	
		Strain JG-3	
	Gram stain	positive	IDA
	Cell shape	Rod	IDA
	Motility	Not reported	IDA
	Sporulation	Not reported	NAS
	Temperature range	<−5 °C to 30 °C	NAS
	Optimum temperature	~20 °C	IDA
	pH range; Optimum	no data; 7	IDA
	Carbon source	R2A, TSA complex media	IDA
MIGS-6	Habitat	Terrestrial, permafrost soil	IDA
MIGS-6.3	Salinity	0-7 % NaCl	IDA
MIGS-22	Oxygen requirement	aerobic	IDA
MIGS-15	Biotic relationship	free-living	IDA
MIGS-14	Pathogenicity	Non-pathogen	NAS
MIGS-4	Geographic location	University Valley, Dry Valleys, Antarctica	IDA
MIGS-5	Sample collection	December, 2009	IDA
MIGS-4.1	Latitude	77d 51.817 s S	IDA
MIGS-4.2	Longitude	160d43.524 s E	IDA
MIGS-4.4	Altitude	37-42 cm below soil surface, in ice-cemented permafrost	IDA

^a^Evidence codes - IDA: Inferred from Direct Assay, TAS: traceable author statement (i.e., a direct report exists in the literature), NAS: Non-traceable Author Statement (i.e., not directly observed for the living, isolated sample, but based on a generally accepted property for the species, or anecdotal evidence). These evidence codes are derived from the Gene Ontology project [17]

**Fig. 2:**
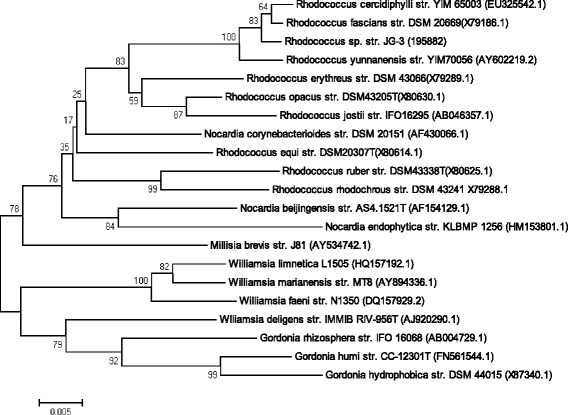
Phylogenetic tree highlighting the position of *Rhodococcus* sp. JG-3 relative to selected taxonomically classified strains within the genus *Rhodococcus* and within the family *Nocardiaceae*. Phylogenetic inferences were obtained using the neighbor-joining method within MEGA6.05 [22]. Numbers at the nodes are percentages of bootstrap values obtained by repeating the analysis 1,000 times to generate a tree using the maximum composite likelihood model. The GenBank accession numbers for the 16S rRNA gene are in parentheses

The 16S rRNA gene sequence of *Rhodococcus* sp. JG-3 was compared using NCBI nucleotide BLAST [18] against the nucleotide collection database (nr/nt) under default parameters, and excluding uncultured microorganisms. *Rhodococcus* sp. JG-3 showed 99 % similarity to that of *R. cercidiphylli* str. BZ22 [19] (GenBank accession: HQ588861.1), a cold adapted isolate from an industrial site contaminated with heavy oil and heavy metals, and which has demonstrated low temperature degradation of petroleum hydrocarbons [9], and 99 % similarity to *Rhodococcus* sp. K4-07B (GenBank accession: EF612291) isolated from a semiarid lead-zinc mine tailing site [20]. Phylogenetic analysis based on the 16S rRNA gene of taxonomically classified type strains of the family *Nocardiaceae* placed *Rhodococcus fascians*DSM 20669 [21] as the closest validly named species to *Rhodococcus* sp. JG-3 (Fig. 2). *R. fascians*DSM 20669 was originally isolated from sweet peas and has an optimum growth temperature of 24 to 27 °C [21].

## Genome sequencing information

### Genome project history

*Rhodococcus sp.* JG-3 was selected for sequencing in 2012 as part of a DOE Joint Genome Institute (JGI) Community Sequencing Program (Quarterly) project to sequence 12 cryophilic isolates from permafrost and cryoenvironments. The Improved Quality Draft assembly and annotation were completed on May 30, 2013. The complete genome sequence of strain JG-3 is available for public access in DDBJ/EMBL/GenBank under accession numbers AXVF01000001- AXVF01000009. The date of Release was December 12, 2013. Table 2 presents the main project information and its association with MIGS version 2.0 compliance [23]. The MIGS record associated with this strain is found in Additional file 1: Table S1.

**Table 2:** Project information

MIGS ID	Property	Term
MIGS 31	Finishing quality	Improved-high-quality draft
MIGS-28	Libraries used	Illumina Std. PE, Illumina Clip PE
MIGS 29	Sequencing platforms	Illumina HiSeq 2000
MIGS 31.2	Fold coverage	1298.1× Ilumina coverage
MIGS 30	Assemblers	AllpathsLG
MIGS 32	Gene calling method	Prodigal, GenePrimp
	Locus Tag	K414
	Genbank ID	AXVF00000000
	GenBank Date of Release	December 12, 2013
	GOLD ID	Gi22490
	BIOPROJECT	PRJNA195882
MIGS 13	Source Material Identifier	ARS Culture collection, NRRL: B-65292)
	Project relevance	Permafrost, adaptation to cold, carbon metabolism

### Growth conditions and DNA isolation

*Rhodococcus* JG-3 was grown to stationary phase on TSB medium at room temperature. Genomic DNA was isolated using the Epicentre MasterPure Gram Positive DNA Purification Kit (Epicentre, Madison, Wisconsin) as per the manufacturer’s instructions. Purified DNA was evaluated with the NanoDrop 1000 (Thermoscientific, Wilmington, Delaware), according to the standards of the DOE Joint Genome Institute.

### Genome sequencing and assembly

The draft genome of *Rhodococcus sp*. JG–3 was generated at the DOE Joint Genome Institute (JGI) using the Illumina technology. An Illumina std shotgun library and long insert mate pair library was constructed and sequenced using the Illumina HiSeq 2000 platform [24]. 20,820,738 reads totaling 3,123.1 Mb were generated from the std shotgun sequence and 41,292,560 reads totaling 3,757.6 Mb were generated from the long insert mate pair library. All general aspects of library construction and sequencing performed at the JGI. All raw Illumina sequence data was passed through DUK, a filtering program developed at JGI, which removes known Illumina sequencing and library preparation artifacts [25]. Filtered Illumina reads were assembled using AllpathsLG (PrepareAllpathsInputs: PHRED 64 = 1 PLOIDY = 1 FRAG COVERAGE = 75 JUMP COVERAGE = 25; RunAllpathsLG: THREADS = 8 RUN = std pairs TARGETS = standard VAPI WARN ONLY = True OVERWRITE = True) [26]. The final draft assembly contained 9 contigs in 6 scaffolds. The total size of the genome is 5.3 Mb. The final assembly is based on 3,122.6 Mb of Illumina Std PE, 3,757.6 Mb of Illumina CLIP PE post filtered data, which provides an average 1298.1X Illumina coverage of the genome.

### Genome annotation

Genes were identified using Prodigal [27], followed by a round of manual curation using GenePRIMP [28] for finished genomes and Draft genomes in fewer than 10 scaffolds. The predicted CDSs were translated and used to search the National Center for Biotechnology Information nonredundant database, UniProt, TIGRFam, Pfam, KEGG, COG, and InterPro databases. The tRNAScanSE tool [29] was used to find tRNA genes, whereas ribosomal RNA genes were found by searches against models of the ribosomal RNA genes built from SILVA [30]. Other non–coding RNAs such as the RNA components of the protein secretion complex and the RNase P were identified by searching the genome for the corresponding Rfam profiles using INFERNAL [17]. Additional gene prediction analysis and manual functional annotation was performed within the Integrated Microbial Genomes platform [1] developed by the Joint Genome Institute, Walnut Creek, CA, USA [31].

## Genome properties

The improved high quality draft genome includes 9 contigs in 6 scaffolds, for a total size of 5286918 bp, 64.41 % GC content. Most of the genome (96 %, 5092715 bp) assembled into one scaffold. For the genome, 5067 genes were predicted, 4998 of which are protein-coding genes; 3977 protein coding genes were assigned to a putative function with the remaining annotated as hypothetical proteins. The properties and statistics of the genome are summarized in Tables 3 and 4.

**Table 3:** Nucleotide content and gene count levels of the genome

Attribute	Value	% of Total
Genome size (bp)	5,286,918	100.00
DNA coding (bp)	4,884,848	92.40
DNA G + C (bp)	3,405,333	64.41
DNA scaffolds	6	100.00
Total genes	5,067	100.00
Protein coding genes	4,998	98.64
RNA genes	69	1.36
Pseudo genes	60	1.18
Genes in internal clusters	NA	
Genes with function prediction	3,977	24.18
Genes assigned to COGs	3,805	75.09
Genes with Pfam domains	4,134	81.59
Genes with signal peptides	370	7.30
Genes with transmembrane helices	1,192	23.52
CRISPR repeats	1	-

**Table 4:** Number of genes associated with general COG functional categories

Code	Value	% age	Description
J	176	4.17	Translation, ribosomal structure and biogenesis
A	1	0.02	RNA processing and modification
K	443	10.50	Transcription
L	173	4.10	Replication, recombination and repair
B	1	0.02	Chromatin structure and dynamics
D	31	0.76	Cell cycle control, Cell division, chromosome partitioning
V	53	1.26	Defense mechanisms
T	213	5.05	Signal transduction mechanisms
M	179	4.24	Cell wall/membrane biogenesis
N	6	0.14	Cell motility
U	43	1.02	Intracellular trafficking and secretion
O	133	3.15	Posttranslational modification, protein turnover, chaperones
C	275	6.52	Energy production and conversion
G	288	6.83	Carbohydrate transport and metabolism
E	380	9.01	Amino acid transport and metabolism
F	97	2.3	Nucleotide transport and metabolism
H	184	4.36	Coenzyme transport and metabolism
I	233	5.52	Lipid transport and metabolism
P	244	5.78	Inorganic ion transport and metabolism
Q	166	3.93	Secondary metabolites biosynthesis, transport and catabolism
R	566	13.42	General function prediction only
S	333	7.89	Function unknown
-	1262	24.91	Not in COGs

The total is based on the total number of protein coding genes in the genome

## Conclusion

The genome sequence of *Rhodococcus* sp. JG-3 will be used for examination of the molecular traits of cold adaptation and to aid understanding of carbon metabolism in cryoenvironments. This is the first reported genome of a bacterium isolated from the Upper Dry Valley permafrost and will provide insight into how microbes survive such extreme conditions. As the availability of genomes from cryophilic strains increases, it may be possible to infer if there is a phylogenetic basis for some cold adaptive traits, as well as identify novel molecular mechanisms for cold adaptation.

## Additional file

Additional file 1: Table S1. Associated MIGS record. (DOC 73 kb)

## References

[1] Goordial J, Lamarche-Gagnon G, Lay C-Y, Whyte L (2013). Left Out in the Cold: Life in Cryoenvironments.

[2] Soina VS, Mulyukin AL, Demkina EV, Vorobyova EA, El-Registan GI (2004). The structure of resting bacterial populations in soil and subsoil permafrost. Astrobiology.

[3] Bottos EM, Vincent WF, Greer CW, Whyte LG (2008). Prokaryotic diversity of arctic ice shelf microbial mats. Environmental microbiology.

[4] Tuorto SJ, Darias P, McGuinness LR, Panikov N, Zhang T, Häggblom MM, Kerkhof LJ (2014). Bacterial genome replication at subzero temperatures in permafrost. The ISME journal.

[5] Goordial J, Whyte L (2014). Microbial Life in Antarctic Permafrost Environments.

[6] Larkin MJ, Kulakov LA, Allen CC (2005). Biodegradation and *Rhodococcus*–masters of catabolic versatility. Current Opinion in Biotechnology.

[7] Haeggblom M, Salkinoja-Salonen M (1991). Biodegradability of chlorinated organic compounds in pulp bleaching effluents. Water Science & Technology.

[8] Whyte LG, Hawari J, Zhou E, Bourbonnière L, Inniss WE, Greer CW (1998). Biodegradation of Variable-Chain-Length Alkanes at Low Temperatures by a Psychrotrophic Rhodococcussp. Applied and environmental microbiology.

[9] Margesin R, Moertelmaier C, Mair J. Low-temperature biodegradation of petroleum hydrocarbons (n-alkanes, phenol, anthracene, pyrene) by four actinobacterial strains. International Biodeterioration & Biodegradation 2012.

[10] Whyte L, Slagman S, Pietrantonio F, Bourbonniere L, Koval S, Lawrence J, Inniss W, Greer C (1999). Physiological adaptations involved in alkane assimilation at a low temperature by *Rhodococcus* sp. strain Q15. Applied and environmental microbiology.

[11] Ruberto LA, Vazquez S, Lobalbo A, Mac CW (2005). Psychrotolerant hydrocarbon-degrading *Rhodococcus* strains isolated from polluted Antarctic soils. Antarctic Science.

[12] Lacelle D, Davila AF, Fisher D, Pollard WH, DeWitt R, Heldmann J, Marinova MM, McKay CP (2013). Excess ground ice of condensation–diffusion origin in University Valley, Dry Valleys of Antarctica: Evidence from isotope geochemistry and numerical modeling. Geochimica et Cosmochimica Acta.

[13] Field D, Garrity G, Gray T, Morrison N, Selengut J, Sterk P, Tatusova T, Thomson N, Allen MJ, Angiuoli SV (2008). The minimum information about a genome sequence (MIGS) specification. Nature biotechnology.

[14] Skerman VBD, McGowan V, Sneath PHA (1980). Approved lists of bacterial names. International Journal of Systematic Bacteriology.

[15] Stackebrandt E, Rainey FA, Ward-Rainey NL (1997). Proposal for a new hierarchic classification system, *Actinobacteria* classis nov. International journal of systematic bacteriology.

[16] Castellani A, Chalmers A (1919). Family *Nocardiaceae*. Manual of Tropical Medicine.

[17] Ashburner M, Ball CA, Blake JA, Botstein D, Butler H, Cherry JM, Davis AP, Dolinski K, Dwight SS, Eppig JT (2000). Gene Ontology: tool for the unification of biology. Nature genetics.

[18] Altschul SF, Gish W, Miller W, Myers EW, Lipman DJ (1990). Basic local alignment search tool. Journal of molecular biology.

[19] Li J, Zhao G-Z, Chen H-H, Qin S, Xu L-H, Jiang C-L, Li W-J (2008). < i > Rhodococcus cercidiphylli</i > sp. nov., a new endophytic actinobacterium isolated from a < i > Cercidiphyllum japonicum</i > leaf. Systematic and applied microbiology.

[20] Mendez MO, Neilson JW, Maier RM (2008). Characterization of a bacterial community in an abandoned semiarid lead-zinc mine tailing site. Applied and environmental microbiology.

[21] Goodfellow M (1984). Reclassification of *Corynebacterium fascians* (Tilford) Dowson in the Genus *Rhodococcus* as *Rhodococcus fascians* comb. nov. Systematic and Applied Microbiology.

[22] Tamura K, Stecher G, Peterson D, Filipski A, Kumar S (2013). MEGA6: Molecular Evolutionary Genetics Analysis Version 6.0. Molecular biology and evolution.

[23] Field D, Garrity G, Gray T, Morrison N, Selengut J, Sterk P, Tatusova T, Thomson N, Allen MJ, Angiuoli SV (2008). The minimum information about a genome sequence (MIGS) specification. Nat Biotechnol.

[24] Bennett S (2004). Solexa ltd. Pharmacogenomics.

[25] Mingkun L CA, Han J. DUK. unpublished 2011.

[26] Gnerre S, MacCallum I, Przybylski D, Ribeiro FJ, Burton JN, Walker BJ, Sharpe T, Hall G, Shea TP, Sykes S (2011). High-quality draft assemblies of mammalian genomes from massively parallel sequence data. Proceedings of the National Academy of Sciences.

[27] Hyatt D, Chen G-L, LoCascio P, Land M, Larimer F, Hauser L (2010). Prodigal: prokaryotic gene recognition and translation initiation site identification. BMC bioinformatics.

[28] Pati A, Ivanova NN, Mikhailova N, Ovchinnikova G, Hooper SD, Lykidis A, Kyrpides NC (2010). GenePRIMP: a gene prediction improvement pipeline for prokaryotic genomes. Nature methods.

[29] Lowe TM, Eddy SR (1997). tRNAscan-SE: a program for improved detection of transfer RNA genes in genomic sequence. Nucleic acids research.

[30] Pruesse E, Quast C, Knittel K, Fuchs BM, Ludwig W, Peplies J, Glöckner FO (2007). SILVA: a comprehensive online resource for quality checked and aligned ribosomal RNA sequence data compatible with ARB. Nucleic acids research.

[31] Markowitz VM, Mavromatis K, Ivanova NN, Chen I-MA, Chu K, Kyrpides NC (2009). IMG ER: a system for microbial genome annotation expert review and curation. Bioinformatics.

