# Supplementary material for: Improved-high-quality draft genome sequence of Rhodococcus sp. JG-3, a eurypsychrophilic Actinobacteria from Antarctic Dry Valley permafrost
Source: Stand Genomic Sci. 2015 Sep 3;10:61. doi: 10.1186/s40793-015-0043-8 (PMC4572675; doi:10.1186/s40793-015-0043-8)
Supplement: Additional file 1: Table S1. — Associated MIGS record. (DOC 73 kb) [file 40793_2015_43_MOESM1_ESM.doc]

Associated MIGS Record

***Table S1.*** *Associated MIGS record*

| **MIGS-ID** | field name | description |
| --- | --- | --- |
| **MIGS-1** | Submit to INSDC/Trace archives |  |
| **1.1** | PID |  |
| **1.2** | Trace Archive |  |
| **MIGS-2** | MIGS CHECK LIST TYPE |  |
| **MIGS-3** | Project Name | Genomic basis of thermal adaption and carbon metabolism in permafrost isolates |
| **MIGS-4** | Geographic Location | University Valley, Antarctica |
| **4.1-4.2** | Latitude and Longitude | 77d 51.817s S, 160d43.524s E |
| **4.3** | Depth | 37–42 cm below surface |
| **4.4** | Altitude | 1650–1800 m.a.s.l |
| **MIGS-5** | Time of Sample collection | December, 2009 |
| **MIGS-6** | Habitat (EnvO) | Terrestrial, permafrost |
| **6.1** | temperature | −20 |
| **6.2** | pH | 7.5 |
| **6.3** | salinity |  |
| **6.4** | chlorophyll |  |
| **6.5** | conductivity |  |
|
| **6.6** | light intensity | subsurface- no light |
| **6.7** | dissolved organic carbon (DOC) | 0.013% |
| **6.8** | current |  |
| **6.9** | atmospheric data |  |
| **6.10** | density |  |
| **6.11** | alkalinity |  |
| **6.12** | dissolved oxygen |  |
| **6.13** | particulate organic carbon (POC) |  |
| **6.14** | phosphate |  |
| **6.15** | nitrate |  |
| **6.16** | sulfates |  |
| **6.17** | sulfides |  |
| **6.18** | primary production |  |
| **MIGS-7** | Subspecific genetic lineage |  |
| **MIGS-9** | Number of replicons |  |
| **MIGS-10** | Extrachromosomal elements |  |
| **MIGS-11** | Estimated Size |  |
| **MIGS-12** | Reference for biomaterial or Genome report |  |
| **MIGS-13** | Source material identifiers |  |
| **MIGS-14** | Known Pathogenicity |  |
|
| **MIGS-15** | Biotic Relationship | free-living |
| **MIGS-16** | Specific Host |  |
| **MIGS-17** | Host specificity or range (taxid) |  |
| **MIGS-18** | Health status of Host |  |
| **MIGS-19** | Trophic Level |  |
| **MIGS-22** | Relationship to Oxygen | aerobic |
| **MIGS-23** | Isolation and Growth conditions | *R2A* |
| **MIGS-27** | Nucleic acid preparation |  |
| **MIGS-28** | Library construction | Illumina Std PE, Illumina Clip PE |
| **28.1** | Library size |  |
| **28.2** | Number of reads | 62113298 |
| **28.3** | vector |  |
| **MIGS-29** | Sequencing method | Illumina HiSeq 2000 |
| **MIGS-30** | Assembly |  |
| **30.1** | Assembly method | AllpathsLG |
| **30.2** | estimated error rate |  |
| **30.3** | method of calculation |  |
| **MIGS-31** | Finishing strategy |  |
| **31.1** | Status | Improved-high-quality draft |
| **31.2** | coverage | 1298.1X Illumina coverage |
| **31.3** | contigs | 9 |
| **MIGS-32** | Gene calling method | Prodigal, GenePrimp |
|  | Genome Database release | JGI IMG database |
|  | GOLD ID | Gi22490 |
|  | Genbank ID | [AXVF00000000](http://www.ncbi.nlm.nih.gov/nuccore/AXVF00000000).1 |
|  | Genbank Date of Release | December 12, 2013 |
|  | Project relevance | Permafrost, adaptation to cold, carbon metabolism |
| **MIGS-33** | Relevant e-resources |  |
|  |  |  |
|  |  |  |
